# Pilocarpine-induced seizures trigger differential regulation of microRNA-stability related genes in rat hippocampal neurons

**DOI:** 10.1038/srep20969

**Published:** 2016-02-12

**Authors:** Erika R. Kinjo, Guilherme S. V. Higa, Bianca A. Santos, Erica de Sousa, Marcio V. Damico, Lais T. Walter, Edgard Morya, Angela C. Valle, Luiz R. G. Britto, Alexandre H. Kihara

**Affiliations:** 1 Núcleo de Cognição e Sistemas Complexos, Centro de Matemática, Computação e Cognição, Universidade Federal do ABC, São Bernardo do Campo, SP, Brazil; 2 Departamento de Fisiologia e Biofísica, Instituto de Ciências Biomédicas, Universidade de São Paulo, São Paulo, SP, Brazil; 3 Programa de Neuroengenharia, Instituto Internacional de Neurociências Edmond e Lily Safra, ISD, Macaíba, RN, Brazil; 4 Laboratório de Neurociências, LIM 01, Departamento de Patologia, Faculdade de Medicina, Universidade de São Paulo, São Paulo, SP, Brazil

## Abstract

Epileptogenesis in the temporal lobe elicits regulation of gene expression and protein translation, leading to reorganization of neuronal networks. In this process, miRNAs were described as being regulated in a cell-specific manner, although mechanistics of miRNAs activity are poorly understood. The specificity of miRNAs on their target genes depends on their intracellular concentration, reflecting the balance of biosynthesis and degradation. Herein, we confirmed that pilocarpine application promptly (<30 min) induces status epilepticus (SE) as revealed by changes in rat electrocorticogram particularly in fast-beta range (21–30 Hz). SE simultaneously upregulated XRN2 and downregulated PAPD4 gene expression in the hippocampus, two genes related to miRNA degradation and stability, respectively. Moreover, SE decreased the number of XRN2-positive cells in the hilus, while reduced the number of PAPD4-positive cells in CA1. XRN2 and PAPD4 levels did not change in calretinin- and CamKII-positive cells, although it was possible to determine that PAPD4, but not XRN2, was upregulated in parvalbumin-positive cells, revealing that SE induction unbalances the accumulation of these functional-opposed proteins in inhibitory interneurons that directly innervate distinct domains of pyramidal cells. Therefore, we were able to disclose a possible mechanism underlying the differential regulation of miRNAs in specific neurons during epileptogenesis.

In the nervous system, control of protein translation by microRNAs (miRNAs) has been recently investigated in distinct situations^1^,^2^, including neural development^3^,^4^,^5^,^6^, neurological disorders^7^,^8^,^9^ and adaptation to distinct environmental situations^10^,^11^,^12^. miRNAs are short nucleotide sequences (20–24 nt), which post-transcriptionally regulate mRNA copy levels and translation efficiency through complementary binding of small stretches of base pairs, typically in the 3′ untranslated region^13^,^14^.

As previously stated, miRNA/mRNA interactions follow probabilistic rather than deterministic operandi^15^. Therefore, specificity of a particular miRNA depends on its cytosolic concentration. In turn, miRNA copy levels are the result of the balance between biosynthesis and degradation. In spite of detailed mechanisms underlying miRNA biogenesis have been reported^16^,^17^,^18^, knowledge about molecules related to miRNA stability and degradation is poor and incipient^19^. Few recent studies disclosed the involvement of 5′–3′ exoribonuclease 2, also known as XRN2, in miRNA degradation, and PAPD4, an atypical poly(A) polymerase, in miRNA stability^20^,^21^.

After induction of status epilepticus (SE), changes in neuronal circuits are driven by changes in gene expression and protein translation^22^,^23^,^24^,^25^. The involvement of miRNAs in this process has been extensively investigated^26^,^27^,^28^. Intriguingly, several reports disclosed that regulation of miRNAs populations during epileptogenesis is cell-29,30 and region-specific^31^. In spite of all efforts, there are clear gaps about how induction of SE mechanistically affects general miRNA activity. In this study, we demonstrated for the first time that the balance of miRNA-stability genes is changed by SE. Moreover, we were able to disclose that functionally-opposed genes XRN2 and PAPD4 are regulated in a cell-specific, region-depending coordinated fashion.

## Materials and Methods

### Ethics Statement

All experiments were carried out with healthy male Wistar rats (*Rattus novergicus*) weighing between 270 and 300 g and mean age ranging from 80–90 days. Experiments with animals were conducted in accordance with guidelines of the NIH and the Brazilian Society for Laboratory Animals. Experimental protocol (#19/2009) was approved by the Ethics Committee in Animal Experimentation of the Institute of Biomedical Sciences/University of São Paulo (ICB/USP). All animals were housed in a vivarium approved by ICB/USP Ethics Committee in Animal Experimentation, with free access to food and water.

### ECoG recordings and pilocarpine-induced status epilepticus (SE)

These procedures were described in detail previously^32^. Briefly, following induction of anesthesia, bipolar 150 μm-diameter nichrome electrodes were implanted bilaterally over neocortical area (AP: −1.5 mm, ML: ±3.0 mm) according to skull references^33^, and after a recovery period of approximately 10 days, ECoG recordings were performed in a 21-channel Nihon–Koden electroencephalograph (Neurofax EEG 4400). After thirty minutes of basal activity recording, animals from pilocarpine-induced SE group (pilocarpine; N = 5) received methyl scopolamine injection (1 mg/kg, s.c.; Sigma-Aldrich, St. Louis, MO, USA) to minimize the peripheral cholinergic effects, followed by pilocarpine hydrochloride (360 mg/kg, i.p.; Sigma-Aldrich) administration to induce generalized seizures, which was continuously recorded for thirty minutes after its initiation. We also obtained recordings from control animals, which received methyl scopolamine injection followed by similar volume of sterile saline instead of pilocarpine (N = 5; data not shown). The same protocol for SE induction was performed and the hippocampi were removed for different methodologies.

### Real-Time PCR

The procedures for Real-time PCR were described in detail previously^34^. Briefly, hippocampi of animals from SE group (N = 8) and from control group (N = 8) were directly extracted and homogenized within 1–1.5 ml TRIzol reagent (Invitrogen, Carlsbad, CA, USA). Total RNA was extracted according to suggested protocol by manufacturer. All Real-time PCR assays were conducted and analyzed by Rotor-Gene 6000 Real-Time Rotary Analyzer (Corbett Robotics Inc., San Francisco, CA) with definite primers for rat XRN2 (forward, 5′-TCGAGGAGGGCGACAGGGAT-3′; reverse, 5′-GGGCGGTGGCAAAGGGTACT-3′) and rat PAPD4 (forward, 5′-ACAGGGTTGTCTACGCCGCC-3′; reverse, 5′-CGCGGGCGTGTTAAGTTGGG-3′). cDNA abundance for cyclophilin A (forward, 5′-GCGTTTTGGGTCCAGGAATGGC-3′; reverse, 5′-TTGCGAGCAGATGGGGTGGG-3′) was determined as internal control. Dissociation curves of PCR products were obtained by heating samples from 60–95 °C, in order to evaluate primer specificity. Relative quantification of target gene expression was evaluated using the comparative CT method as previously described in detail^35^,^36^. Values were entered into T-Test, with the significance level set at 5%.

### Subcellular fractionation and immunoblotting

Hippocampi of animals from control group (N = 8) and from SE group (N = 8) were rapidly dissected, washed with phosphate buffered saline (PBS) and homogenized in H buffer for protein isolation of subcellular compartments for immunoblotting analysis. Homogenates from tissues diluted in H buffer (0.32 M sucrose, 4 mM HEPES and protease inhibitors, pH 7.4) were serially centrifuged to separate intracellular compartments, as briefly described: for 5 min at 750 G, 4 °C, to isolate the nuclear fraction (pellet); for 10 min at 9000 G, 4 °C, to isolate mitochondrial fraction (pellet; not evaluated); and for 30 min at 20000 G, 4 °C to separate plasma membrane (pellet) from the supernatant containing the endoplasmic reticulum (not evaluated) and cytosol. This method was adapted from^37^,^38^. Protein concentration was determined by the BCA method (# 23225, Thermo Scientific, Rockford, IL, USA) and bovine serum albumin was used as the standard, following manufacturer protocol. Proteins in the membrane preparations were separated by sodium dodecyl sulfate-polyacrylamide gel electrophoresis (SDS-PAGE; 10% gel) and transferred to nitrocellulose membranes. Blots were incubated with 5% non-fat milk in TBST buffer for 2 h at room temperature to block nonspecific binding of the antibodies. After rinsed in TBST, blots were incubated with rabbit anti-PAPD4 (1:1,000; #ab103884, Abcam). After the primary antibody incubation, blots were rinsed in TBST and incubated with goat anti-rabbit-peroxidase (ECL kit; Amersham, Buckinghamshire, England) for 2 h at room temperature. Detection of labeled proteins was achieved by using the enhanced chemiluminescent system (ECL kit; Amersham). Measurements of optical densities (OD) were performed using ImageJ software (National Institute of Mental Health, Bethesda, Maryland, USA). OD of the bands were normalized using the mean value of nuclear and cytosolic compartments found for the control group and the OD value found for the total protein loading given by the Ponceau S staining. For statistical analyses, data from four independent experiments were entered into a two-way ANOVA.

### Immunohistochemistry

The procedures for immunohistochemistry were described in detail earlier^34^. Briefly, brains of animals from SE group (N = 4) and from control group (N = 4) were collected and fixed for 8 hours in 1% paraformaldehyde (PFA) in phosphate buffer 0.1 M pH 7.3 (PB), and cryoprotected in 30% sucrose solution for at least 48 hours at 4 °C. Coronal sections (12 μm) containing hippocampus were incubated overnight with primary antibodies against XRN2 (1:200; #ab72181, Abcam) or PAPD4 (1:200; #ab103884, Abcam) at room temperature. For double labeling experiments, we used mouse anti-CamKIIα (1:300; #ab22609, Abcam), mouse anti-calretinin (1:500; #MAB1568, Millipore, Billerica, MA, USA) and mouse anti-parvalbumin (1:500; #P3088, Sigma-Aldrich). The fluorescent secondary antibodies used were anti- rabbit IgG tagged to Alexa 488 (1:200–1:500, Invitrogen) and anti-mouse conjugated to Alexa-546 and -647 (1:200–1:500, Invitrogen). Controls for the experiments consisted of the omission of primary antibodies; no staining was observed in these cases. 4′,6-diamidino-2-phenylindole (DAPI) was diluted in the same incubation solution of the secondary antibodies to counter-stain the brain sections. After washing, the tissue was mounted using Vecta Shield (Vector Labs, Burlingame, CA, USA), and analyzed in Nikon TS100F inverted microscope (Nikon Instruments Inc., Melville, NY, USA) or Leica DM 5500 (Leica Microsystems, Germany). Figures were mounted with Adobe Photoshop CS5. Manipulation of the images was restricted to brightness and contrast adjustments of the whole image.

### Image quantification

Image analyses were performed with ImageJ software (National Institute of Mental Health, Bethesda, Maryland, USA) and NIS elements (Nikon Instruments Inc.), as previously described^32^,^34^,^39^. We employed approximately 25 hippocampal slices from 4 animals (4–6 hippocampal slices from each animal). After channel separation (RGB) of color images, we performed quantification of the number of XRN2/PAPD4-positive cells, followed by division by the number of nuclei. For quantification of PAPD4 labeling in subcellular compartments and double-labeling experiments, quantification of the brightness-weighted average of delimited areas was performed. In these cases, area of interest (AOI) was defined by the labeling of one channel, and analysis was performed in another channel, as for instance, labeling of XRN2 and PAPD4 in the green channel, defined by DAPI/CamKIIα/PV/CR labeling in the blue or red channel. Each value was normalized by the mean pixel intensity of all labeled XRN2 or PAPD4-positive cells of the correspondent slice. Values from all analyses were exported to Excel (Microsoft, Redmond, WA, USA) and entered into T-Test, with significance level set at 5%. Images and charts were prepared using Adobe Photoshop CS5 (Adobe Systems Inc., San Jose, CA, USA).

## Results

### Pilocarpine-induced SE

**Figure 1:**
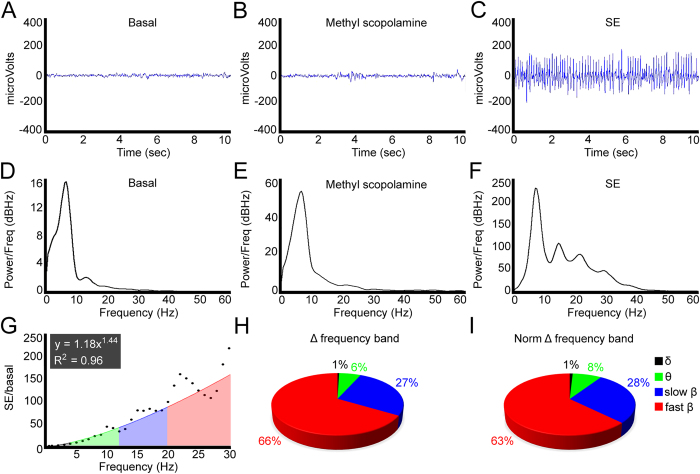
Pilocarpine-induced status epilepticus (SE). Representative local field potentials of an animal submitted to 30 minutes of pilocarpine-induced SE. (**A**,**B**) Basal and methyl scopolamine recordings presented similar electrophysiological characteristics. (**C**) After treatment with pilocarpine, an overall increase of the neuronal electrical activity was observed. (**D**,**E**) As expected, power spectrum of basal and methyl scopolamine periods showed similar frequency composition. (**F**) After pilocarpine-induced activity, remarkable differences in the frequency bands took place, particularly at the range of fast beta oscillations (FBO). (**G**) Changes in the frequency ranging from 0.5–30 Hz relative to the basal are showed in the graph. A power function adjusted to the data points clearly indicates enhancement of the highest frequencies, especially of the fast beta (21–30 Hz). (**H**) Using the equation shown in (**G**) and considering the values that define the frequency bands as the intervals for the integral, we estimated the changes of the SE activity relative to the basal. Notably, a pronounced contribution of the high beta oscillations was established, representing 66% of the total. (**I**) In order to normalize the integral values plotted in (**H**) we divided them by the respective interval that compose the frequency range (i.e. δ: 4.5; θ: 7; slow β: 9 and fast β: 10). These data confirmed that fast beta oscillations compose the most regulated frequency range in this SE model (63%).

In order to demonstrate that pilocarpine leads to the establishment of epileptiform activity, we performed electrocorticographic (ECoG) recordings during the induction of SE. The fraction of animals that presented pilocarpine-induced SE was around 95%, considering all the animals used for the different methodologies. Figure 1A–C shows the representative ECoG relative to the conditions named basal, methyl scopolamine and pilocarpine-induced SE. In the basal and methyl scopolamine conditions, we observed low frequency and small amplitude electrical activity. Pilocarpine generated an overall increase of the electrical activity, consistent with epileptiform discharges. To further analyze the consequences of pilocarpine-induced SE, we conducted power spectra analysis of the evaluated conditions (Fig. 1D–F). We observed a general increase in the power of all frequencies, but particular alterations in the beta frequency range. To estimate the contribution of the frequency bands after pilocarpine-induced SE, we determined the ratio of each frequency from 0.5 to 30 Hz of this condition relative to the basal (Fig. 1G). Computational analysis revealed that the data points fit with a power function, and the trendline generated by this equation showed intensification of the highest frequencies, particularly of fast beta oscillations (FBO, 21–30 Hz). Taking the limits that classify the frequency ranges as the intervals for the integral calculation, we determined the participation of each band of the induced experimental condition relative to the basal (Fig. 1H). This analysis substantiated the hypothesis that pilocarpine treatment increased the highest oscillations, considering the distribution of the values δ: 1%; θ: 6%; low β: 27% and high β: 66%. As the frequency ranges encompass distinct intervals (δ: 4.5; θ: 7; low β: 9 and high β: 10), we normalized the integral values by these intervals (Fig. 1I). This analysis confirmed the larger contribution of the high beta frequency after pilocarpine-induced SE, taking into account the values δ: 1%; θ: 8%; low β: 28% and high β: 63%.

### SE differentially affects XRN2 and PAPD4 gene expression

**Figure 2:**
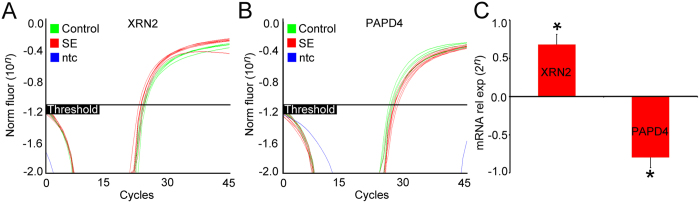
Quantification of XRN2 and PAPD4 gene expression in the hippocampus after induction of SE. (**A**) Amplification plots of quantitative real time PCR from control (green) and SE (red) hippocampal samples using primers designed for XRN2 gene. Notice that SE samples require a lesser amount of cycles to emerge when compared with control samples. (**B**) Amplification plots from control (green) and SE (red) hippocampal samples using primers designed for PAPD4 gene. SE samples need additional cycles to emerge when compared with control samples. (**C**) XRN2 and PAPD4 expression in SE samples normalized with control. SE increases XRN2 gene expression (2^0.679 = 1.601 fold-expression, *P* < 0.01) and decreases PAPD4 gene expression (2^0.799 = 0.574 fold-expression, *P* < 0.01). *P < 0.01 *vs.* controls in T-Test.

By using primers specifically designed for XRN2 and PAPD4 we generated amplification plots from cDNA serial dilutions to ascertain these efficiencies. Amplification plots indicated that pilocarpine-induced SE increased XRN2 transcript levels compared to the control group (2^0.679 = 1.601 fold-expression, *P* < 0.01), while reduced PAPD4 mRNA levels were observed in the hippocampus of SE animals (2^0.799 = 0.574 fold-expression, *P* < 0.01) compared to control animals. Cyclophilin gene expression was used as internal control (Fig. 2A-C).

### SE reduces the number of XRN2 and PAPD4-positive cells in a region-specific manner

**Figure 3:**
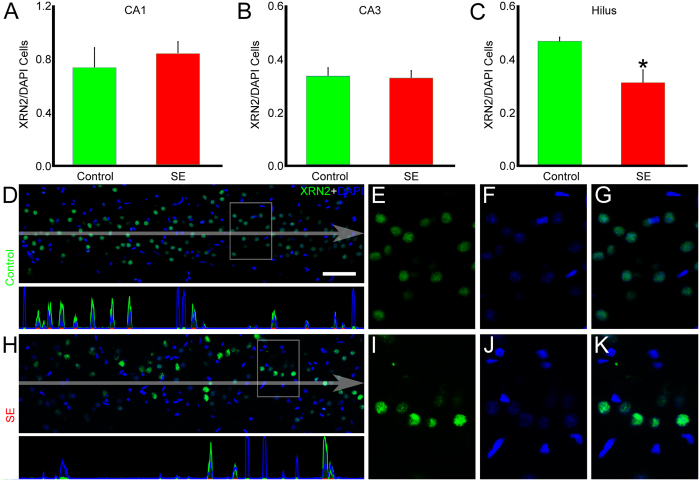
Quantification of XRN2-positive cells after SE induction. In order to verify whether the induction of SE promoted changes in the number of XRN2-positive cells (green), we performed immunofluorescence experiments in coronal sections of rats from control and SE groups counter-stained with DAPI (blue). (**A**–**C**) Quantification of XRN2-positive cells relative to the total number of nuclei showed no differences in CA1 and CA3 areas of the SE group, compared to controls, whereas a significant decrease was observed in the hilus (*P* < 0.05). (**D**) In the representative image of the hilus of DG of a control animal, it is possible to visualize that XRN2 concentrates in the nuclear compartment of most of the cells. Pixel intensity profile demonstrates high spatial correlation of both cell markers. (**E**–**G**) In high magnification of a selected area, it is possible to see the overlaying of both cell markers. (**H**) In the representative image of the SE group, we observed diminished number of XRN2-positive cells. Pixel intensity profile shows decreased spatial correlation between green and blue channels. (**I**–**K**) In high magnification of a representative area, we observed that the number of nuclei that accumulate XRN2 is reduced when compared to controls. Bars represent standard errors of mean. *P < 0.05 in T-Test. Scale bar: 50 μm.

**Figure 4:**
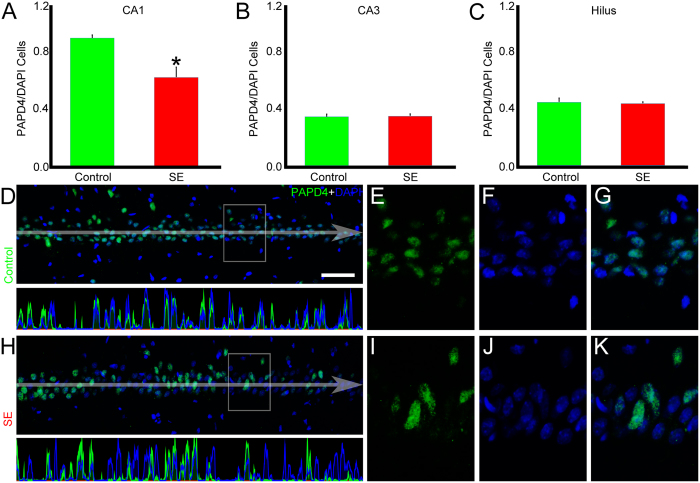
Quantification of PAPD4-positive cells after SE induction. To examine the distribution of PAPD4 (green) after SE induction, we performed immunofluorescence experiments in coronal sections of rats from control and SE groups counter-stained with DAPI (blue). (**A**–**C**) Quantitative analysis of the number of PAPD4-positive cells relative to the number of nuclei demonstrated a significant reduction in CA1 (*P* < 0.05), while we were not able to detect changes in CA3 and hilus of DG. (**D**) In the representative image of CA1 of a control animal, we can see that most of the nuclei are labeled with PAPD4. Pixel intensity profile demonstrates high spatial correlation of both cell markers. (**E**–**G**) In high magnification of a representative area, it is possible to see the overlapping of both cell markers. (**H**) In the representative image of the SE group, it is shown reduced number of PAPD4-positive cells. Pixel intensity profile indicates decreased spatial correlation between green and blue channels. (**I**–**K**) In high magnification of a selected area, we observed reduction in the number of PAPD4-positive cells compared to the controls. Bars represent standard errors of mean. *P < 0.05 in T-Test. Scale bar: 50 μm.

Once we determined that XRN2 mRNA was changed after SE induction, we examined whether specific regions of the hippocampus showed differences in the number of XRN2-positive cells. Quantification of the XRN2-positive cells relative to the number of nuclei in control and in SE groups revealed that the amount of cells expressing this protein remained stable in CA1 (0.74 ± 0.14 *vs.* 0.84 ± 0.08, respectively) and CA3 (0.34 ± 0.03 *vs.* 0.33 ± 0.03, respectively) (Fig. 3). On the other hand, a significant decrease was observed in the hilus comparing control and SE groups (0.47 ± 0.01 *vs.* 0.31 ± 0.05, respectively, *P* < 0.05). The same analysis applied to PAPD4 showed reduction in the number of PAPD4-positive cells relative to the quantity of nuclei stained by DAPI comparing control and SE groups in CA1 (0.96 ± 0.02 *vs.* 0.67 ± 0.07, respectively, *P* < 0.05) (Fig. 4), but not in CA3 (0.37 ± 0.02 *vs.* 0.38 ± 0.01, respectively) and hilus (0.48 ± 0.03 *vs.* 0.47 ± 0.01, respectively).

### SE induction does not change PAPD4 accumulation in specific subcellular compartments

**Figure 5:**
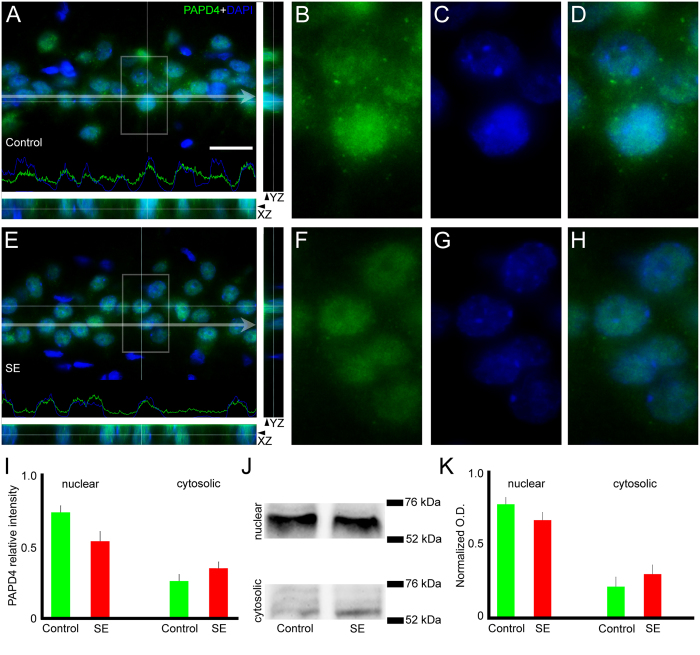
SE does not change the accumulation of PAPD4 in subcellular compartments. To examine whether the induction of SE altered the subcellular distribution of PAPD4 (green), we performed immunofluorescence experiments in coronal sections of rats from control and SE groups counter-stained with DAPI (blue). ((**A**) upper) In the representative image of CA1 of a control animal, it is possible to observe PAPD4 labeling in the nuclei and cytosol. Pixel intensity profile demonstrates the green signal (PAPD4) inside the blue signal (revealing nuclear localization) and also in areas where the blue-delimited areas is virtually absent, demonstrating its cytoplasmic distribution as well. ((**A**) lower, right) Images shown are z-axis analysis to evidence the PAPD4 labeling in both nuclei and cytoplasm. (**B**–**D**) In high magnification of selected area, it is possible to observe the presence of PAPD4 overlaying and surrounding nuclei. ((**E**) upper) In the representative image of CA1 of a SE animal, the same pattern of subcellular distribution of PAPD4 is visualized compared to controls. Pixel intensity profile shows the green line (PAPD4) inside the blue signal and also in areas where the blue-delimited areas is virtually absent. ((**E**) lower, right) Representative images of z-axis analysis confirmed PAPD4 immunostaining in the nuclei and cytosol of cells. (**F**–**H**) In high magnification of representative areas, it is possible to see that induction of SE did not change the amount of PAPD4 in the cellular compartments. (**I**) Quantification of the mean pixel intensity confirmed the stable accumulation of PAPD4 in the nuclei and cytoplasm after SE induction. (**J**) Optical band densities of nuclear and cytosolic subcellular compartments from control and SE groups, normalized by the total protein loading in the corresponding lane to the 52–76 kDa molecular weight range. (**K**) We were not able to observe significant differences in PAPD4 protein levels in the nuclear and cytosolic fractions of controls and SE animals. Bars represent standard errors of mean. Scale bar: 20 μm.

Once PAPD4 was previously described as having nuclear and cytosolic distribution according to the maturation stage of the cell^34^, we aimed to verify whether SE could change the accumulation of the protein in these cellular compartments. Comparing control and SE groups, mean pixel analysis revealed that the intensity labeling of PAPD4 in the nuclear (0.73 ± 0.04 *vs.* 0.54 ± 0.07) and cytoplasmic (0.27 ± 0.04 *vs.* 0.35 ± 0.04) compartments did not significantly change in the hippocampus (Fig. 5). To confirm these data, we performed subcellular fractionation of the hippocampus of control and SE groups followed by immunoblotting analysis. When compared to controls, PAPD4 protein levels remained stable in the hippocampus after induction of SE in both nuclear (0.78 ± 0.05 *vs.* 0.67 ± 0.05, respectively) and cytoplasmic (0.22 ± 0.06 *vs.* 0.31 ± 0.06, respectively) compartments.

### XRN2 and PAPD4 accumulation do not change in excitatory cells after SE induction

**Figure 6:**
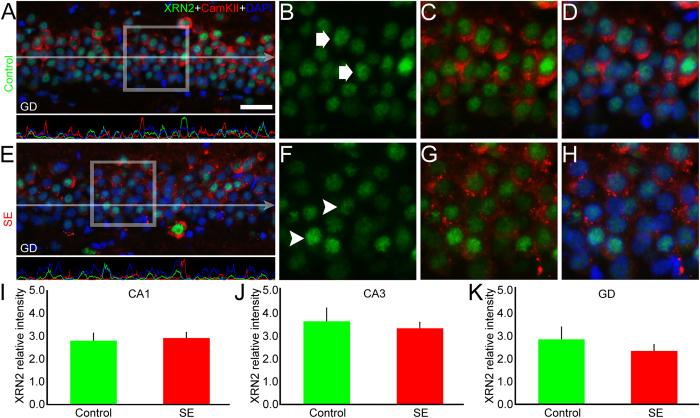
XRN2 accumulation does not change in excitatory neurons after SE induction. In order to examine whether SE induction changes the accumulation of XRN2 (green) in specific neuronal subpopulations, we conducted double-labeling experiments using anti-CamKIIα (red) in coronal sections of control and SE animals counter-stained with DAPI (blue). (**A**) In the representative image of the DG it is possible to observe that XRN2 is present in CamKIIα-positive granule cells of the control group. The pixel intensity profile reveals that the green signal is surrounded by the red signal, demonstrating the presence of XRN2 in excitatory neurons of the control group. (**B**–**D**) In high magnification of selected area, we confirmed the presence of XRN2 in CamKIIα- positive cells (white arrows) of the DG. (**E**) We performed the same analysis in the SE group. The pixel intensity profile revealed the same pattern observed in the control group. (**F**–**H**) In high magnification of selected area it is possible to observe that the amount of XRN2 in CamKIIα-positive neurons (white arrowheads) is similar to what we observed in the control group. (**I**–**K**) Quantification of the mean pixel intensity of XRN2 in CamKIIα-positive neurons of the control group did not show modifications after SE induction in CA1, CA3 and dentate granule cells, respectively. Bars represent standard errors of mean. Scale bar: 25 μm.

**Figure 7:**
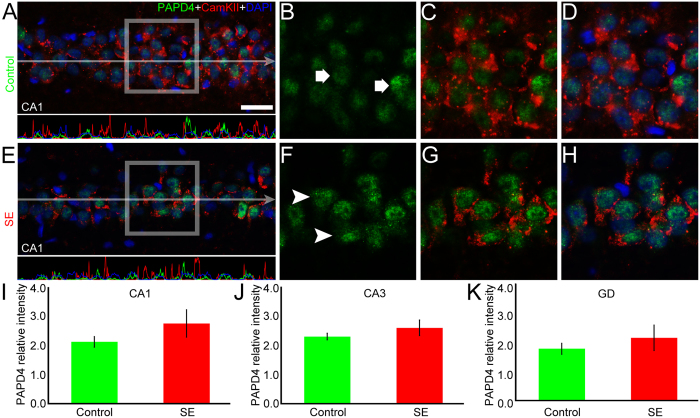
SE does not affect the amount of PAPD4 in excitatory neurons. We also performed double-labeling experiments to verify whether SE induction changes PAPD4 (green) levels in excitatory neurons of the hippocampus, using anti-CamKIIα (red) in coronal sections of control and SE animals counter-stained with DAPI (blue). (**A**) In the representative image of CA1, we visualized PAPD4 in CamKIIα-positive pyramidal cells. Pixel intensity profile demonstrates the red signal overlays the green one, showing the presence of PAPD4 in pyramidal cells. (**B**–**D**) In high magnification of representative area, we confirmed the presence of PAPD4 in CamKIIα- positive cells (white arrows) of CA1. (**E**) The same analysis conducted in the SE group showed that the amount of PAPD4 in pyramidal cells is similar to what we found in the control animals. The pixel intensity profile confirmed the same pattern observed in the control group. (**F**–**H**) In high magnification of selected area, it is possible to see that PAPD4 levels remained stable in excitatory neurons (white arrowheads) after SE induction compared to the control group. (**I**–**K**) Quantification of the mean pixel intensity of PAPD4 in excitatory cells revealed no significant changes after SE induction in CA1, CA3 and granule cells of DG, respectively. Bars represent standard errors of mean. Scale bar: 25 μm.

In order to address the pattern of XRN2 and PAPD4 in principal cell layers, we performed double-labeling experiments using anti-CamKIIα, a marker for excitatory neurons^40^,^41^. Comparing control and SE groups, our analysis revealed that the intensity labeling of XRN2 remained stable in pyramidal cells of CA1 (2.79 ± 0.33 *vs.* 2.90 ± 0.26), CA3 (3.56 ± 0.57 *vs.* 3.26 ± 0.26, respectively) and in the granule cell layer (2.76 ± 0.52 *vs.* 2.26 ± 0.27, respectively) (Fig. 6). Additionally, when compared to control animals, we were not able to detect differences in the mean pixel intensity of XRN2 in CamKIIα-positive cells in the hilus of SE group (2.71 ± 0.45 *vs.* 2.81 ± 0.18, respectively) (Fig. S1, supplementary information). The same analysis performed for PAPD4 immunolabeling did not reveal changes in the mean pixel intensity in the pyramidal and granular cells of controls compared to SE group in CA1 (2.11 ± 0.18 *vs.* 2.74 ± 0.47, respectively), CA3 (2.29 ± 0.11 *vs.* 2.59 ± 0.26, respectively) and GD (1.77 ± 0.19 *vs.* 2.14 ± 0.44, respectively) (Fig. 7). The same analysis performed in the hilus of control and SE groups did not detect changes in the accumulation of PAPD4 in CamKIIα-positive cells (2.01 ± 0.11 *vs.* 2.24 ± 0.20, respectively) (Fig. S1, supplementary information).

### XRN2 levels remain stable in parvalbumin-positive GABAergic interneurons

**Figure 8:**
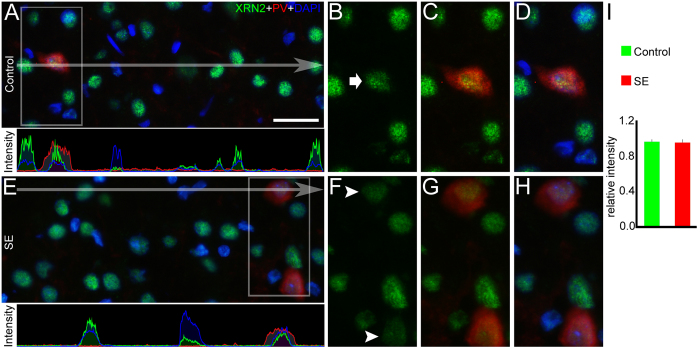
XRN2 levels are stable in parvalbumin (PV)-positive cells after induction of SE. To continue the evaluation of whether the expression of XRN2 (green) is changed in specific neuronal subpopulations after SE induction, we performed double-labeling experiments using anti-PV (red) in coronal sections of control and SE animals counter-stained with DAPI (blue). (**A**) In the representative image of the hilus of DG it is possible to see that XRN2 is present in PV-positive cells of the control group. In the pixel intensity profile, we could observe that the green signal overlays the red signal, demonstrating the co-expression of XRN2 and PV. (**B**–**D**) In high magnification of selected areas, we observed that XRN2 accumulates in PV-positive cells (white arrow). (**E**) We performed the same analysis in the SE group. The pixel intensity profile revealed the same pattern observed in the control group. (**F**–**H**) In high magnification of representative areas, it is possible to visualize that induction of SE did not modify the amount of XNR2 in PV-positive cells (white arrowheads) when compared to PV-positive cells of the control group. (**I**) Quantification of XRN2 in PV-positive cells of the SE group relative to the PV-positive cells of the control group confirmed the stable accumulation of this protein after SE induction. Bars represent standard errors of mean. Scale bar: 25 μm.

In order to assess the pattern of XRN2 accumulation in specific neuronal subpopulations after SE induction, we conducted double-labeling experiments using anti-parvalbumin (PV), a calcium binding protein that accumulates in a subset of fast spiking GABAergic interneurons^42^. The mean pixel analysis showed that the intensity labeling of XRN2 in PV-positive cells is very similar comparing controls and SE group (0.96 ± 0.02 *vs.* 0.96 ± 0.03, respectively) (Fig. 8). To further examine the regulation of XRN2 in specific cells, we also performed experiments using anti-calretinin (CR). The mean pixel analysis indicated a stable labeling intensity of XRN2 in CR-positive cells in control and SE groups (0.88 ± 0.12 *vs.* 0.78 ± 0.11, respectively) (Fig. S2, supplementary information).

### SE changes the accumulation of PAPD4 in parvalbumin-positive cells in a region-dependent manner

**Figure 9:**
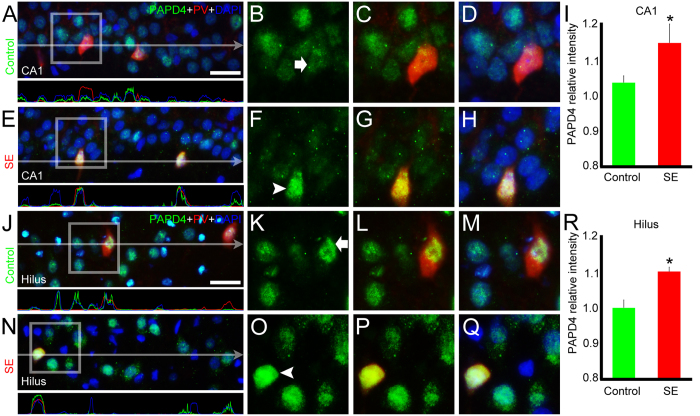
SE increases PAPD4 accumulation in parvalbumin (PV)-positive cells in a region-dependent manner. We also conducted double-labeling experiments using anti-PV (red) to examine the accumulation of PAPD4 (green) in this specific neuronal subpopulation in coronal sections of control and SE animals counter-stained with DAPI (blue). (**A**) In a representative image of CA1, we could observe the presence of PAPD4 in PV-positive cells of the controls. The pixel intensity profile confirmed overlapping of the green and red signals. (**B**–**D**) In high magnification of selected area, it is possible to see accumulation of PAPD4 in PV-positive (white arrow) cells. (**E**) The same analysis was performed in the SE group. As indicated by the pixel intensity profile, PAPD4 accumulates in PV-positive cells, since the green signal overlays the red signal. (**F**–**H**) In high magnification of a representative area, we observed increased accumulation of PAPD4 in PV-positive cells (white arrowhead) when compared to PV-positive cells of controls. (**I**) Quantification of PAPD4 intensity in CA1 confirmed the increased amount of PAPD4 in PV-positive cells of the SE group compared to the PV-positive cells of the control group. (**J**) In a representative image of the hilus of a control animal, it is also possible to observe the PAPD4 labeling in PV-positive cells. In the pixel intensity profile, we see an overlap between the green and red signals, evidencing that PV-positive cells accumulate PAPD4. (**K**–**M**) In high magnification of selected area, we observed PAPD4 immunolabeling in PV-positive (white arrow) cells. (**N**) We performed the same analysis in the SE animals. The pixel intensity profile showed PAPD4 accumulation in PV-positive cells, in agreement with the overlay of the green and red lines. (**O**–**Q**) In high magnification of selected area, we observed higher intensity labeling of PAPD4 in PV-positive cells (white arrowhead) than in PV-positive cells of controls. (**R**) Quantification of PAPD4 pixel intensity in the hilus endorsed its higher accumulation in PV-positive cells of the SE group compared to the PV-positive cells of the control animals. Bars represent standard errors of mean. *P < 0.05 in T-Test. Scale bar: 25 μm.

Following the same analysis performed for XRN2, we conducted double-labeling experiments using anti-PV to verify whether PAPD4 amount is altered in PV-containing GABAergic interneurons after SE induction. Comparing control and SE groups, mean pixel intensity analysis revealed increased accumulation of this protein in PV-positive cells after SE both in CA1 (1.04 ± 0.02 *vs.* 1.15 ± 0.05, respectively, *P* < 0.05) and in the hilus (1.00 ± 0.02 *vs.* 1.10 ± 0.01, respectively, *P* < 0.05) (Fig. 9), demonstrating a particular regulation of PAPD4 in specific GABAergic interneurons which is region-dependent, since we were not able to observe difference in CA3 and DG areas (data not shown). We also performed double-labeling experiments using anti-CR in order to assess the regulation of PAPD4 in this specific subtype of interneurons. We were not able to detect changes in the mean pixel intensity analysis, revealing a stable accumulation of PAPD4 in CR-positive cells of control and SE groups (1.01 ± 0.05 *vs.* 1.07 ± 0.07, respectively) (Fig. S3, supplementary information).

## Discussion

The control of transcriptome in neurons is finely regulated by miRNAs, since these cells operate under a wide variety of conditions, from health to disease. In the nervous system, control of gene expression by miRNAs has been investigated in fundamental physiological processes, including activity-dependent changes that trigger the molecular mechanisms of neuronal plasticity^1^,^43^,^44^,^45^. Since every miRNA regulates the translation of hundreds of proteins, it is now becoming clear that these molecules are involved in the molecular-driven changes necessary for physiological processes such as memory and learning^11^,^12^, as well as in pathological conditions as epilepsy^31^,^46^,^47^.

In this study, we employed pilocarpine-induced SE, which has been used as a model for induction of temporal lobe epilepsy^48^,^49^. We were able to detect increase in all analyzed frequency ranges, but an overwhelming contribution of fast-beta oscillations. Interestingly, XRN2 and PAPD4 gene expression were differentially regulated by SE. Moreover, since both genes have opposite-driven actions in miRNA stability, it was surprising that changes in this balance take place in specific hippocampal regions, such as hilus of DG and CA1. In fact, recent studies combining multiple *in vivo* recordings and mathematical modeling started to disclose detailed contribution of specific areas of hippocampus in superficial EEG recordings^50^,^51^,^52^. In spite of these findings, it is well-established that GABAergic inhibitory neurons are responsible for multiple roles in the neural networks^53^, including the control of the electrical activity of large neuronal populations^54^, determination of frequency of action potentials in target neurons^55^ and generation of fast network oscillations in cortical circuits^56^,^57^.

Taking into account these essential roles, it was remarkable that changes in miRNA-stability related proteins were verified specifically in PV-positive neurons, a GABAergic neuronal cell type that preferentially accumulates PAPD4 in hippocampus^34^, in a region-dependent manner. Therefore, extended miRNA half-life and increased widespread activity would be expected, considering the catalytic role of this cytoplasmic poly(A) polymerase responsible for the 3′-terminal adenylation of both pre-miRNA and miRNA^20^. Our results indicated that changes in protein accumulation in specific neuronal subtypes did not match with gene expression of both XRN2 and PAPD4. Indeed, as the hippocampal cell population is vast and heterogeneous^58^,^59^, it was not surprising that the protein amount in specific cells did not correspond to XRN2 and PAPD4 mRNA levels from the whole hippocampal homogenates due to methodological limitations. It is possible that the transcript levels of XRN2 and PAPD4 reflect changes in other cell types that were not addressed in this study. Additionally, several mechanisms may underlie the independent regulation of mRNA and protein levels, including complex, intricate control of transcription by miRNAs^60^. Other studies addressed the issue and presented possibilities for the lack of association between mRNA and protein levels, such as stability of proteins, half-life of different proteins and less variability of mRNA and also posttranscriptional and post-translational regulation, including the participation of miRNAs^61^.

Finally, these evidences indicated that regulation of the transcriptome in pivotal inhibitory interneurons may take part of the molecular-driven plasticity associated with the control of network activity in physiological processes^62^,^63^, for example memory and learning, whereas abnormal influence may impact in changes observed in diseases, such as Alzheimer’s^64^,^65^ and epilepsy^66^,^67^.

Epileptogenesis triggers regulation of gene expression and protein translation, leading to reorganization of hippocampal networks. In this process, elegant studies revealed that changes in miRNAs levels occur in specific manner, depending on the subtype and location of neurons^29^,^30^,^31^. In spite of these efforts, the basis underlying the control of miRNA activity is unknown. In this regard, our results disclosed that miRNA-stability related genes XRN2 and PAPD4 constitute a possible mechanism underlying the differential regulation of miRNAs in specific neurons during epileptogenesis.

## Additional Information

**How to cite this article**: Kinjo, E. R. *et al.* Pilocarpine-induced seizures trigger differential regulation of microRNA-stability related genes in rat hippocampal neurons. *Sci. Rep.* **6**, 20969; doi: 10.1038/srep20969 (2016).

## Supplementary Material

Supplementary Information
