# Supplementary material for: Pilocarpine-induced seizures trigger differential regulation of microRNA-stability related genes in rat hippocampal neurons
Source: Sci Rep. 2016 Feb 12;6:20969. doi: 10.1038/srep20969 (PMC4751485; doi:10.1038/srep20969)
Supplement: Supplementary Information [file srep20969-s1.doc]

**Pilocarpine-induced seizures trigger differential regulation of microRNA-stability**

**related genes in rat hippocampal neurons.**

Erika R. Kinjo, Guilherme S. V. Higa, Bianca A. Santos, Erica de Sousa, Marcio V.

Damico, Lais T. Walter, Edgard Morya, Angela C. Valle, Luiz R. G. Britto &

Alexandre H. Kihara


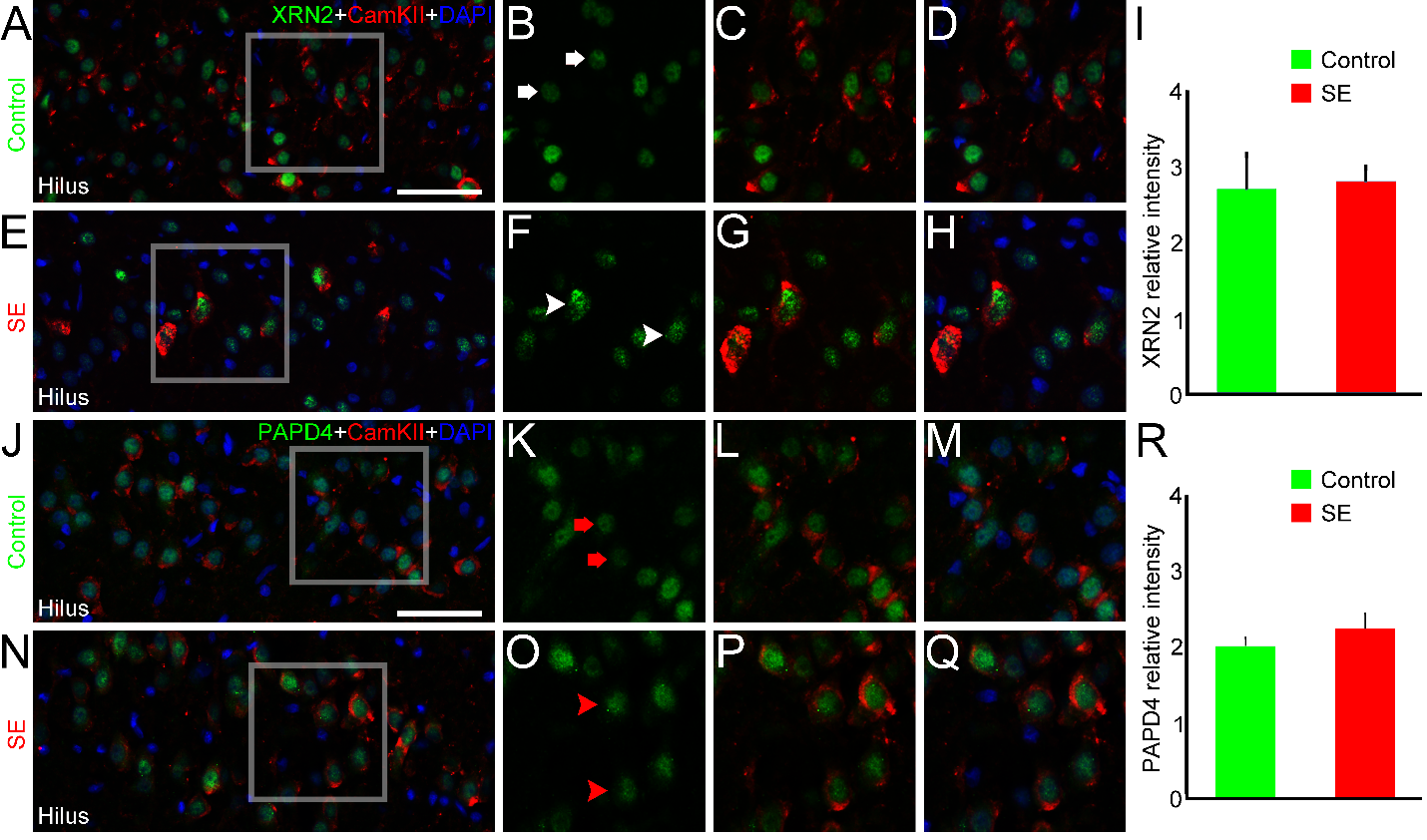


**Supplementary figure S1. XRN2 and PAPD4 levels do not change in excitatory cells of the**

**hilus of DG after status epilepticus (SE) induction.** (A) In the representative image of the

hilus, it is possible to see that XRN2 (green) staining is present in CamKIIα (red) -positive cells

in coronal sections of the control group counterstained with DAPI (blue). (B-D) In high magnification of a selected area, we confirmed the presence of XRN2 in excitatory neurons of

the hilus of controls (white arrows). (E) The same analysis was employed for the SE group. (F-H) In high magnification of representative area, it is possible to see that the amount of XRN2 in

excitatory cells of the hilus (white arrowheads) from controls is similar to that observed in SE

animals. (I) Quantification of the mean pixel intensity of XRN2 in CamKIIα-positive cells in the

hilus. We were not able to detect significant changes comparing control and SE groups. (J) We

conducted the same analysis for PAPD4 (green). In the representative image of the hilus, it is

possible to observe the accumulation of PAPD4 in CamKIIα-positive cells of the control group.

(K-M) In high magnification of a selected area, we confirmed the presence of PAPD4 in excitatory neurons of the hilus of the control group (red arrows). (N) The same analysis was

performed in the SE group. (O-Q) In high magnification of selected area, it is possible to see that

SE induction did not modify the amount of PAPD4 in CamKIIα-positive cells (red arrowheads).

(R) Quantification of the mean pixel intensity of PAPD4 in CamKIIα-positive cells in the hilus.

We were not able to detect significant changes comparing control and SE groups. Bars represent

standard errors of mean. Scale bar: 50 µm.


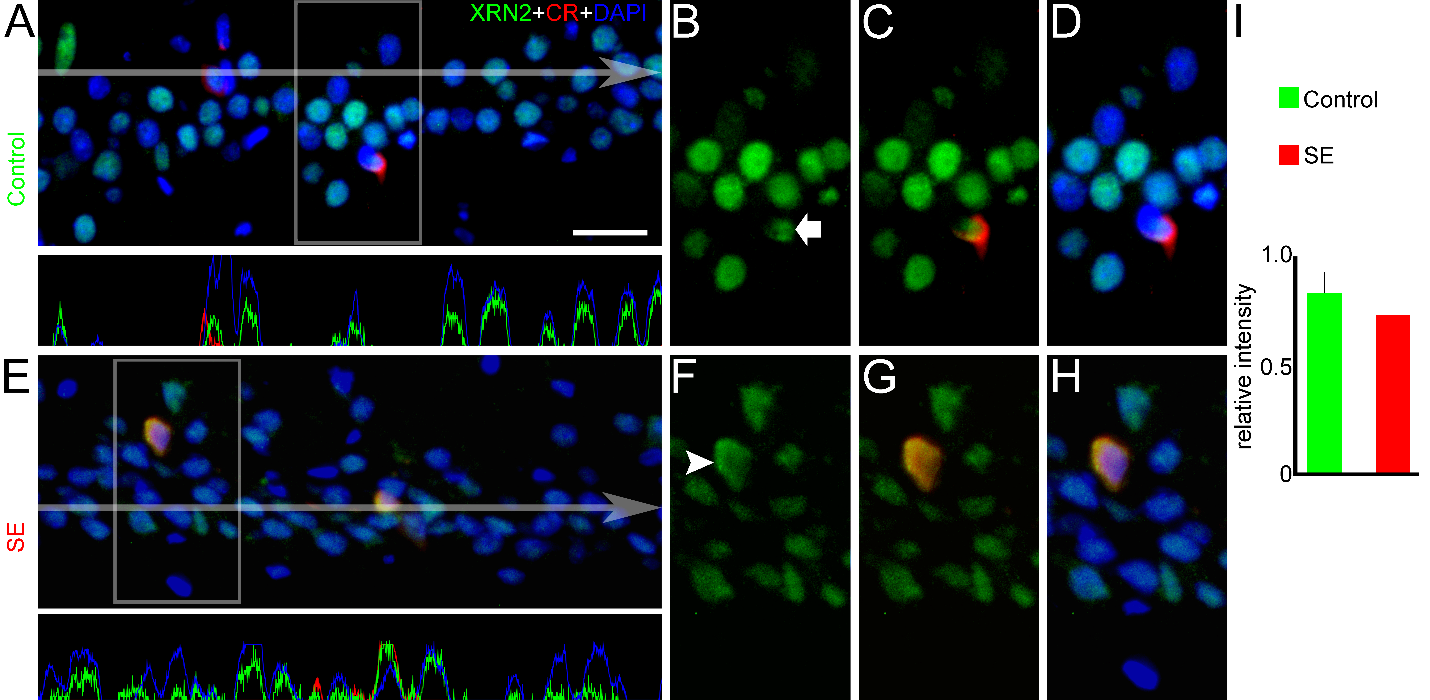


**Supplementary figure S2. XRN2 levels are stable in calretinin (CR)-positive cells after**

**induction of SE.** In order to verify whether the expression of XRN2 (green) is changed in

specific subpopulations of interneurons after SE induction, we performed double-labeling

experiments using anti-CR (red) in coronal sections of control and SE animals counter-stained

with DAPI (blue). (A) In the representative image of CA1 it is possible to see that XRN2 is present in CR-positive cells of the control group. In the pixel intensity profile, we could observe

that the green signal overlays the red signal, demonstrating the co-expression of XRN2 and CR.

(B-D) In high magnification of selected areas, we observed accumulation of XRN2 in CR

positive cells (white arrow). (E) We performed the same analysis in the SE group. The pixel

intensity profile revealed the same pattern observed in the control group. (F-H) In high

magnification of representative areas, it is possible to visualize that induction of SE did not

modify the amount of XNR2 in CR-positive cells (white arrowhead) when compared to CR-positive cells of the control group. (I) We were not able to detect significant differences in the

quantification of XRN2 in CR-positive cells from controls compared to the levels of XRN2 in

CR-positive cells of the SE group. Bars represent standard errors of mean. Scale bar: 25 µm.


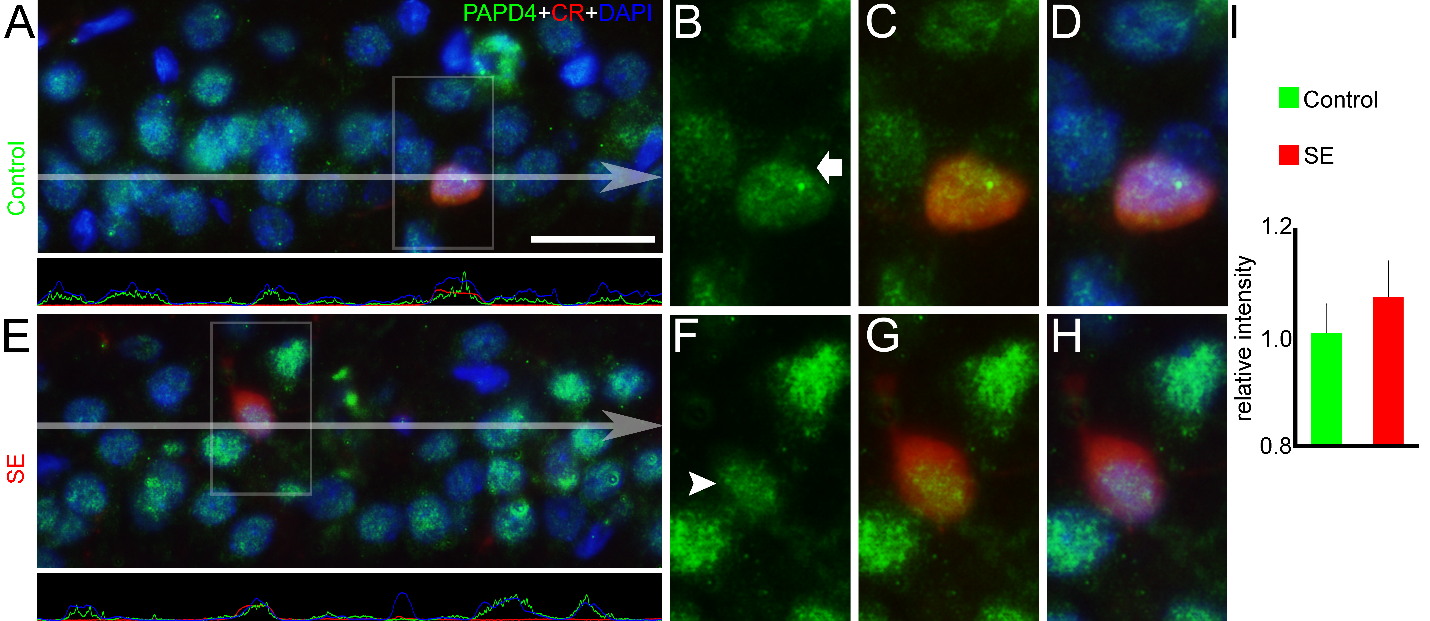


**Supplementary figure S3. PAPD4 levels do not change in calretinin (CR)-positive cells after**

**induction of SE.** We also conducted double-labeling experiments using anti-CR (red) to

examine the expression of PAPD4 (green) in this specific subpopulation of interneurons in

coronal sections of control and SE animals counter-stained with DAPI (blue). (A) In a representative image of CA1, we could observe the presence of PAPD4 in CR-positive cells of

the controls. The pixel intensity profile confirmed an overlapping of the green and red signals.

(B-D) In high magnification of selected area, it is possible to see that CR-positive (white arrow)

cells accumulate PAPD4. (E) The same analysis was performed in the SE group. As indicated by

the pixel intensity profile, PAPD4 is expressed in CR-positive cells, since the green signal overlays the red signal. (F-H) In high magnification of a representative area, we did not observe

differences in accumulation of PAPD4 in CR-positive cells (white arrowhead) when compared to

CR- positive cells. (I) We were not able to detect significant differences in the quantification

of PAPD4 in CR-positive cells of controls compared to the levels of PAPD4 in CR-positive cells

of the SE group. Bars represent standard errors of mean. Scale bar: 25 µm.


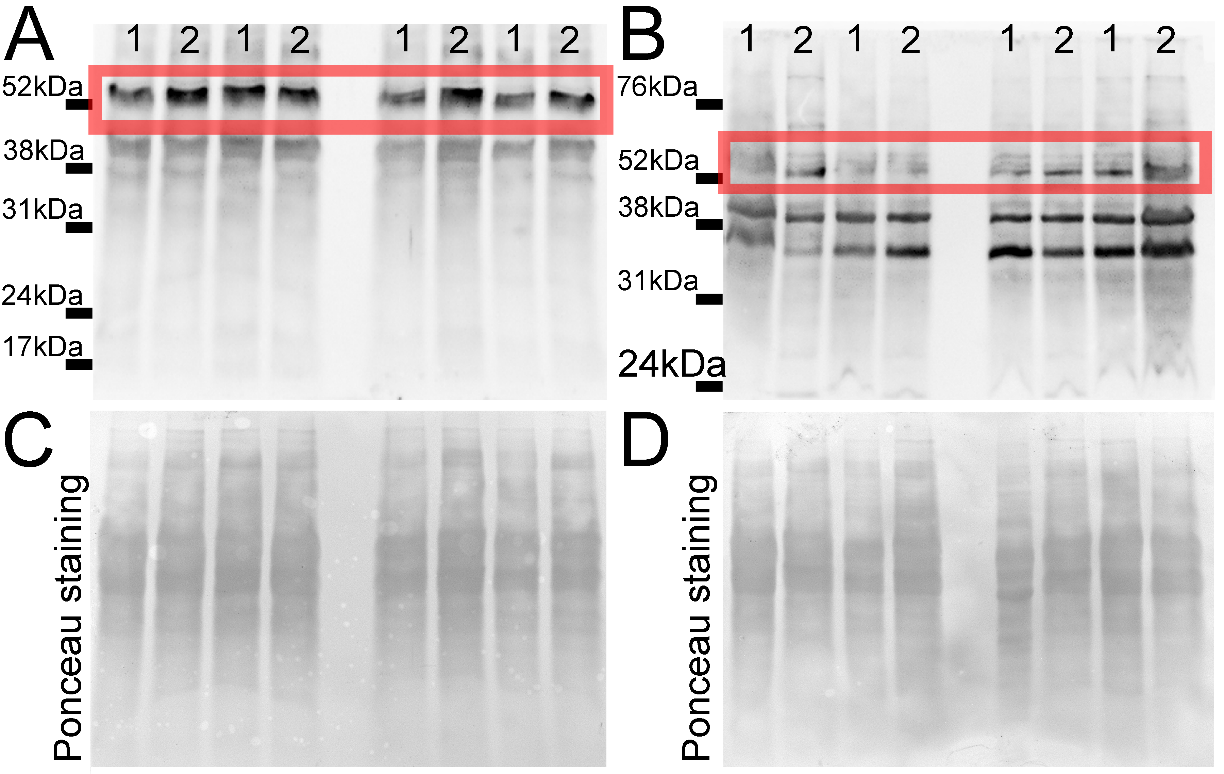


**Supplementary figure S4. SE induction does not change PAPD4 accumulation in specific subcellular compartments.** (A) Uncropped images of western blots of PAPD4 from nuclear compartment from control (lanes 1) and SE animals (lanes 2). (B) Uncropped images of western

blots of PAPD4 from cytosolic compartment from control (lanes 1) and SE animals (lanes 2). Molecular weight markers are represented at right of each image. Red boxes indicate the bands used for quantification of PAPD4 levels. (C, D) Uncropped images of Ponceau S staining from the membranes of nuclear and cytosolic compartments represented above, respectively.
